# Ultrasonic-assisted green, eco-friendly, and economical aqueous extraction of Piperine from pepper species: an alternative to replace organic harmful solvents

**DOI:** 10.1016/j.ultsonch.2026.107986

**Published:** 2026-07-30

**Authors:** Rizwan Ahmad, Deema Alaswad, Ayat Jaffer Alshehab, Mohammed Aldholmi, Ahad Al Sulays, Wala’a Al-Sulais, Aljawharah Alqathama, Fatimah Taiseer Alghorab, Hawraa Ayesh Alibrahim

**Affiliations:** a Department of Natural Products, College of Pharmacy, Imam Abdulrahman Bin Faisal University, 31441, Dammam, Kingdom of Saudi Arabia; b Department of Pharmaceutical Sciences, Faculty of Pharmacy, Umm Al-Qura University, Makkah 21955, Kingdom of Saudi Arabia

**Keywords:** Piperine, USE, Aqueous solutes, UPLCMSMS, Black pepper

## Abstract

**Background:**

Piperine (PPN), a bioactive alkaloid from pepper species, possesses diverse pharmacological properties; however, its poor aqueous solubility impedes its therapeutic potential. The current study aims to develop a green, cost-effective, and eco-friendly aqueous solute-based extraction method for PPN to replace the organic hazardous solvents.

**Methodology:**

Fourteen different solutes were selected and evaluated at various concentrations to determine the optimal concentration for an aqueous solute with maximal solubility for PPN. For the extraction of PPN from pepper species, ultrasound-assisted extraction (USE) was used, whereas for quantification, a green UPLCMSMS (ultra-high pressure liquid chromatography with mass spectrometry) method was developed and validated.

**Results:**

The in-house developed green UPLCMSMS method revealed an accuracy of 95–105 ± 14.24%, r^2^-0.99, limit of detection (0.07–10.3 ± 3.53 ppb), and limit of quantification (0.2–31.21 ± 10.69 ppb) in the linearity range of 0.01–500 ppb. The aqueous solutes of Pluronic F-68 (PF) and ethyl oleate (EO) exhibited superior solubilizing potential of 4235.33 ppb and 3405.24 ppb for PPN, respectively. This suggests a 7x (PF) and 5x (EO) greater solubility compared to water. The small-scale application of the developed model in real-world samples of pepper species revealed the highest PPN content (147.20 ppb) for *P. nigrum* (white), followed by black, long, and cubeb species. The statistical models of one-sample and paired samples t-tests exhibited significant differences (*P < 0.05*) for PPN across the fourteen different solutes and pepper species evaluated.

**Conclusion:**

The study presents a novel, green, and cost-effective aqueous-solutes-based extraction method for PPN, offering a sustainable alternative to hazardous organic solvents commonly used in the pharmaceutical and nutraceutical industries.

## 1 Introduction

Piperine (PPN), an alkaloid belonging to the family of Piperaceae, is predominantly isolated from Piper species, with *Piper nigrum* being the major source. [1], [2] PPN exhibits an array of biological and pharmacological activities such as antioxidant, antihypertensive, antitumor, anti-asthmatic, and antipyretic effects [3], [4], attributed mainly to the presence of an enriched phytochemical profile including alkaloids, phenols, steroids, flavonoids, lignans, and neo-lignans. The chemical diversity with pharmacological activities favors the incorporation of PPN into various pharmaceutical and food products [1], [5]. Despite the promising potential, PPN suffers from the drawback of poor aqueous solubility, limiting its clinical applications where oral dosage form results a decreased bioavailability. [6] This hinders the industrial-scale applications for PPN. Moreover, the aqueous-based PPN formulations displayed incompatibilities with different formulation solutes [7], necessitating the urgency for a sustainable, safer, and non-toxic alternative where the PPN solubility is enhanced without any compromise for biocompatibility. Previous investigations have examined the application of excipients and hydrotropic agents to facilitate the aqueous extraction of PPN. These studies suggest that elevated concentrations of solubilizing agents may augment extraction efficiency, primarily by promoting bulk solubilization and enhancing cell permeabilization. In addition, the analytical method herein signals a gap for a more reliable technique. [8] Concurrently, ultrasound-assisted extraction has seen extensive use for PPN recovery across diverse organic solvent systems to enhance extract yield. [9], [10], [11], [12], [13], [14] Nevertheless, limitations endure, and a significant portion of these methods relies on either organic solvents or relatively high concentrations of solubilizing agents. More importantly, there is less focus on systematic optimization of the solute concentrations, particularly when operating under sub-critical aggregation conditions. Likewise, the integration of solute-assisted aqueous systems with ultrasound extraction has not been thoroughly investigated, and the mechanistic understanding of solute-PPN interactions in such systems remains inadequate. Consequently, there remains a discernible need for the development of an extraction strategy that requires a low solute concentration and combines controlled mechanistic solubilization with process intensification techniques like USE. The USE has seen extensive application in phytochemical isolation; however, a majority of the published methodologies continue to employ organic, consequently restricting their congruence with the tenets of green chemistry. [15], [16], [17] The solute-based techniques have shown substantial promise in augmenting the solubility of hydrophobic compounds to explore sub-CMC and pre-micellar aggregation states. [18], [19], [20] This specific mechanistic aspect remains inadequately investigated within the context of natural product extraction, especially concerning molecules like PPN. Consequently, a cohesive strategy that amalgamates solute-assisted aqueous solubilization, optimized concentration control, and ultrasound-mediated extraction into an efficient, unified workflow is currently absent. Addressing this deficiency, the current study presents an integrated methodology. This approach is founded on a systematic screening of various amphiphilic solutes across a range of concentrations, which is then combined with USE and an environmentally adaptive UPLCMSMS analysis. To make it distinct from conventional methods, the current system utilizes aqueous-solute-based extraction medium, not only to reduce dependence on potentially hazardous organic solvents but also to improve PPN solubility through specific solute-drug interactions. Furthermore, this work intends to offer fresh perspectives on the contribution of sub-CMC solute dynamics to the extraction of hydrophobic natural products. Previous investigations into PPN extraction have largely focused on organic solvent systems or modified ultrasonic-assisted extraction techniques; however, comprehensive exploration of fully aqueous solubilization strategies remains relatively limited. [21], [22]This study aims to develop an integrated approach to PPN aqueous solubilization that is green, eco-, and human-friendly, for different solutes (polymers, surfactants, and solubilizers). A range of solutes at varying concentrations will be evaluated to determine the optimal ratio for solutes: PPN, attaining maximum solubility. To track PPN solubility, an in-house UPLCMSMS method will be developed and validated. The UPLCMSMS-based optimized PPN: solute ratio, will be employed to determine the PPN amount in a real-world black pepper sample, extracted with a high-power USE. The sole purpose of this integrated approach is to establish a green, sustainable, scalable, and biocompatible formulation strategy for PPN. This integrated approach constitutes a departure from solvent-driven extraction, transitioning towards solute-engineered aqueous systems, which in turn offers a scalable and environmentally sustainable alternative for the extraction of natural products.

## 2 Materials and methods

### 2.1 Solutes, standards, instruments, and solvents

The study utilized various solutes (polymers, surfactants, and solubilizers); beta-cyclodextrin, ethyl oleate (UFC Biotechnology), Labrafil M−1944CS, Labrafil M−2125CS, Lauroglycol (FCC-1), polyethylene glycol-2000 (AK scientific), polyvinyl alcohol-MW25000 (Polysciences, Inc.), pluronic F-68 (Gibco), sodium dodecyl sulfate (Promega), sodium alginate (Sigma Aldrich), triton X-100 (Loba Chemie), Tween-20 and −80 (VWR), and 2-polyacrylic acid (Bonnymans). The standard drug Piperine (PPN) was obtained from Rx-Biosciences (USA). For water (18.2 MΩ·cm), an in-house distillation unit from Millipore, USA, was utilized. The extraction process involved a high-frequency (20 kHz) ultrasonic dismembrator (Fisher Scientific) using a probe and transducer generating high-energy waves of 20 kHz. Analysis was carried out using UPLCMSMS (ultra-high pressure liquid chromatography coupled with mass detector) from Shimadzu, Japan (UPLCMSMS-8050). The LCMS-grade solvent (≥99.9%) of ethanol (EtOH) from Merck (Darmstadt, Germany) was used for the chromatographic analysis of PPN.

### 2.2 Development and validation of green UPLCMSMS analytical method

#### 2.2.1 Standard and stock solutions preparation

The standard drug for Piperine (PPN ≥ 98) was dissolved in EtOH to obtain a stock solution of 1 mg/1mL. The stock solution was further diluted to prepare a final working standard of 1 ppm of the PPN concentration. For the development of a calibration curve, eight different linearity ranges (0.01–500 ppb) points were prepared from the working standard of PPN, consisting of 0.01, 0.1, 1, 5, 10, 50, 100, and 500 ppb. All the samples were diluted in HPLC-grade EtOH and filtered through a 0.2 μm syringe filter.

#### 2.2.2 Development of analytical method

For UPLCMSMS method development, the chromatography was carried out using the green solvent of EtOH. The separation was performed on different columns using a mobile phase of H_2_O (A) and EtOH (B). A set of different flow rates (0.1–0.5 mL/min) with injection volumes (1–10 μL) under different column oven temperatures (25-40°C) was evaluated.

The fragmentations for the PPN in the MS were tested using both the + ve and –ve modes of ionization using the ESI interface (electrospray ionization technique). The EtOH solvent was utilized in various ratios of 30%, 50%, and 70% of the mobile phase for the fragmentations with high intensities of the respective fragments (*m*/*z*). The collision energies for the Q1 and Q3 pre-bias were optimized, and based on the peak shape as well as intensities of the PPN fragments, the optimized method was developed for the chromatographic separation of PPN.

#### 2.2.3 Validation of analytical method

The method for PPN analysis was validated using the protocols reported for drug development. [23] The method was checked for accuracy, coefficient of determination r^2^-value, limit of detection (LOD), and limit of quantification (LOQ). The regression equation for the calibration curve was calculated in the linearity range of 0.01–500 ppb, as shown in Table 1.

**Table 1:** Method development and validation parameters for the ppn analytical method.

**UPLCMSMS MDMV parameters**	
**LC conditions**	
Mobile phase	Water (A)2%: Ethanol (B)98%
Flow rate	0.5 mL/min
Retention time	1.3 min
Runtime	2 min (including equilibration time)
Linearity range	0.01–500 ppb
Column dimensions	ThermoFisher Scientific Hypersil GOLDTM column [RP C-18, 50X4mm; 3 mm]
**MS conditions**	
Interface	ESI
Mode	Positive(+ve)
Gas flow	Nebulizing gas (3 L/min); heating and drying gas (10 L/min)
Temperatures	Interface (300°C); heat block (400°C); DL (250°C)
Fragmentation (*m*/*z*)	286.05 to 201.0 to 143.10 to 115.10 (MRM-mode)
Q1 and Q3 Pre bias (V)	201.0 (−20.0 and −20.0), 143.10 (−14.0 and −14.0), 115.10 (−15.0 and −20.0)
Collision energies (CE)	(201) −20, (143.1) −30.9, (115.1) −50
Dwell time (msec)	100
Event time (sec)	0.309
**Regression analysis**	
Accuracy	95–105 ± 14.24%
r^2^-value	0.999
LOD (ppb)	0.07–10.3 ± 3.53
LOQ (ppb)	0.2–31.21 ± 10.69
Regression equation	y = 36801.2X + 226,230
Fragments (*m*/*z*) for CC and quantification of unknown samples	201.0 (base peak)

### 2.3 Selection of optimal aqueous solutes

#### 2.3.1 Screening of the solutes

This study tested fourteen frequently employed solutes: tween-20 (T20), tween-80 (T80), labrafil-M (L19), labrafil-M (L21), lauroglycol (LG), beta-cyclodextrin (CD), polyacrylic acid (PAA), ethyl oleate (EO), polyvinyl alcohol (PVA), sodium alginate (SA), pluronic (PF), triton (TX), sodium dodecyl sulfate (SDS), and poly ethylene glycol (PEG). The reference values (CMC; critical micelle concentration) for the solutes were extracted from the literature: T20 (0.034–0.060 mM), T80 (0.013–0.027 mM), L19 (0.218% v/v), L21 (10%), LG (0.1, 0.5–1 mM), CD (1.5% w/w), PAA (1–10%), EO (15 mg), PVA (0.25–1 mM), SA (1–10% w/v), PF (2–3 mM), TX (0.1–1%w/w), SDS (0.008 mM), and PEG (0.008–0.025 mM). The solutes with detailed information are provided in Table 2.

**Table 2:** Solutes with codes, reference values collected from literature, and the concentrations for finalizing ppn solubility.

	**Solutes**	**Code**	**Reference value**	**Solutes range tested**	**Code**	**Final concentration**	**Optimal concentration**
1	Tween-20	T20	0.034–0.060 mM	1, 3, 5,10,15 (mL)	T20-1	0.5 mL	0.5 mL
					T20-2	1 mL	
					T20-3	2 mL	
2	Tween-80	T80	0.013–0.027 mM	0.5, 2, 4, 8, 10 (mL)	T80-1	0.1 mL	0.1 mL
					T80-2	0.5 mL	
					T80-3	1 mL	
3	Labrafil-M (1944-CS)	L19	0.218% (v/v)	0.05, 0.2, 0.4, 1, 2 (mL)	L19-1	0.01 mL	0.01 mL
					L19-2	0.05 mL	
					L19-3	0.1 mL	
4	Labrafil-M (2125-CS)	L21	10%	0.05, 0.2, 0.4, 1, 2 (mL)	L21-1	0.01 mL	0.01 mL
					L21-2	0.05 mL	
					L21-3	0.1 mL	
5	Lauroglycol (FCC-1)	LG	0.1, 0.5, 1 mM	0.005, 0.02, 0.08, 0.1, 0.5 (mL)	LG-1	0.001 mL	0.001 mL
					LG-2	0.005 mL	
					LG-3	0.01 mL	
6	Beta-cyclodextrin	CD	1.5% w/w	150, 300, 500, 1000, 15,000 (mg)	CD-1	100 mg	100 mg
					CD-2	150 mg	
					CD-3	200 mg	
7	Polyacrylic acid	PAA	1, 5, 10%	20, 40, 80, 100, 200 (mg)	PAA-1	10 mg	10 mg
					PAA-2	20 mg	
					PAA-3	30 mg	
8	Ethyl oleate	EO	15 mg	0.005, 0.02, 0.08, 0.1, 0.5 (mL)	EO-1	0.001 mL	0.001 mL
					EO-2	0.005 mL	
					EO-3	0.01 mL	
9	Polyvinyl alcohol (MW-25000)	PVA	0.25–1 Mm	5, 20, 50, 80, 100 (g)	PVA-1	1 g	1 g
					PVA-2	5 g	
					PVA-3	10 g	
10	Sodium alginate	SA	1, 5, 10% (w/v)	250, 600, 800, 1000, 1500 (mg)	SA-1	100 mg	100 mg
					SA-2	250 mg	
					SA-3	500 mg	
11	Pluronic (F-68)	PF	2, 2.50, 3 Mm	0.5, 2, 4, 8, 10 (mL)	PF-1	0.1 mL	0.1 mL
					PF-2	0.5 mL	
					PF-3	1 mL	
12	Triton (X-100)	TX	0.1, 0.5, 1% (w/w)	1, 2, 5, 10, 15 (mL)	TX-1	0.5 mL	0.5 mL
					TX-2	1 mL	
					TX-3	1.5 mL	
13	Sodium dodecyl sulfate	SDS	0.008 Mm	100, 200, 300, 400, 500 (mg)	SDS-1	50 mg	50 mg
					SDS-2	100 mg	
					SDS-3	150 mg	
14	Poly ethylene glycol (MW-2000)	PEG	0.008, 0.015, 0.025 mM	1, 2, 3, 4, 5 (g)	PEG-1	0.5 g	0.5 g
					PEG-2	1 g	
					PEG-3	1.5 g	

#### 2.3.2 Solubility profile for the solutes

The solutes were dissolved in water using a range of five different concentrations for each solutes: T20 (1, 3, 5, 10, 15 mL), T80 (0.5, 2, 4, 8, 10 mL), L19 (0.05, 0.2, 0.4, 1, 2 mL), L21 (0.05, 0.2, 0.4, 1, 2 mL), LG (0.005, 0.02, 0.08, 0.1, 0.5 mL), CD (150, 300, 500, 1000, 15000 mg), PAA (20, 40, 80, 100, 200 mg), EO (0.005, 0.02, 0.08, 0.1, 0.5 mL), PVA (5, 20, 50, 80, 100 g), SA (250, 600, 800, 1000, 1500 mg), PF (0.5, 2, 4, 8, 10 mL), TX (1, 2, 5, 10, 15 mL), SDS (100, 200, 300, 400, 500 mg), and PEG (1, 2, 3, 4, 5 g).

#### 2.3.3 Selection of the optimal concentration for the solutes

The solutes with more clarity and lack of turbidity (from 2.3.2) were further studied at three different concentrations on an individual basis: T20 (0.5, 1, 2 mL), T80 (0.1, 0.5, 1 mL), L19 (0.01, 0.05, 0.1 mL), L21 (0.01, 0.05, 0.1 mL), LG (0.001, 0.005, 0.01 mL), CD (100, 150, 200 mg), PAA (10, 20, 30 mg), EO (0.001, 0.005, 0.01 mL), PVA (1, 5, 10 g), SA (100, 250, 500 mg), PF (0.1, 0. 5, 1 mL), TX (0.5, 1, 1.5 mL), SDS (50, 100, 150 mg), and PEG (0.5, 1, 1.5 g). The three-level range for the solutes sorted the decisive optimal concentration for individual solutes to be employed for the solubilization of PPN.

### 2.4 Piperine vs optimal aqueous solute concentration

#### 2.4.1 Solubility of PPN in aqueous solutes

A reference solution for the standard drug PPN (1 mg/1mL) was prepared in EtOH to compare (for clarity, turbidity, and visible particles) with the PPN dissolved in aqueous solutes. The optimal aqueous solute concentration (section 2.3.3) for individual solutes (1 mL) was taken in plastic tubes (propylene tubes with caps and 10 mL capacity), added with PPN (1–5 mg/mL), sonicated (30 min), vortexed (10 min), and centrifuged (10,000 rpm for 10 min) to observe the clarity of the individual solute solution. The solubility for PPN (1, 2, 3, 4, 5 mg/mL) was tested in an incremental pattern, where each solute was prepared and repeated in five different batches for the different PPN concentrations to dissolve.

#### 2.4.2 Level-I and II solute concentrations for PPN extraction

This step tested two different levels of concentration for the optimal solutes, exhibiting more amount of PPN at the lowest concentrations of the solutes. Briefly: Level-I evaluated three levels for solute concentrations [low (Conc-I), optimal (Conc-II), and higher (Conc-III)], and the solutes with the lowest concentration showing more PPN amount were studied further in Level-II. The concentrations for Level-II were selected towards more dilution of the optimal Level-I concentrations, to determine the endpoint for enhancement in PPN solubility with a decrease in solute concentration. The lowest solute value with more PPN was diluted further at three concentrations, and PPN solubility was determined.

#### 2.4.3 UPLCMSMS analysis for PPN quantification

The solutions batches with a finalized PPN amount and aqueous solute concentrations for all fourteen solutes were filtered through a 0.2 μm syringe filter and diluted properly to achieve a concentration of 100 ppb for analysis using UPLCMSMS. All fourteen samples were appropriately vortexed and subjected to the in-house developed UPLCMSMS analysis to observe the PPN solubility behavior presented by the individual solutes. In addition, an aqueous PPN solution was prepared to test the PPN solubility in water. Likewise, a reference ethanolic solution was prepared for the standard PPN drug to compare the results for the aqueous solutes vs aqueous PPN solubility. All three different medium PPN solutions (standard solution, aqueous solution, and aqueous solute solutions) were analyzed via the in-house developed UPLCMSMS analytical method.

### 2.5 Applicability of the aqueous-solutes-based PPN-extraction model

#### 2.5.1 USE conditions

The extraction model for PPN employed the previously developed, validated, and reported ultrasonic technique with slight modifications. [24] The USE conditions consisted of: 40% amplitude, 40 s:5s pulse rate, 3 min extraction time, 200 mg sample amount, and a solute volume of 10 mL. Before starting the experimental procedure, the dry pepper seeds were milled under controlled conditions to ensure a uniform particle size. The powder was sieved using a mesh/sieve (stainless steel sieve with apertures of 1.0 mm, obtained from Laboratory test sieve, BS 410–1, Endecotts Ltd., London, England. Subsequently, extraction was carried out utilizing a chosen solvent system. Throughout this process, temperature was maintained under stringent control using a calibrated thermostatic system to minimize experimental variability. In order to mitigate the variability imposed by the particle size and morphology, the powdered material was sieved systematically before extraction.

#### 2.5.2 Aqueous extraction of PPN using the optimal solutes

To evaluate the applicability of the developed aqueous-solutes-based extraction model, PPN was extracted using four different types of the PPN species, namely: *Piper nigrum* black (PN) and white (PW), *Piper longum* (PL), and *Piper cubeb* (PC). The optimal solutes were used to extract PPN using the aforementioned USE conditions. The extracted samples were syringe filtered, centrifuged, and diluted to the desired UPLCMSMS concentrations for further quantification of PPN in these samples.

#### 2.5.3 Two-level extraction of the PPN using the optimal solute concentration

The lowest concentration of the solutes with more PPN extraction was studied further at two different levels (lowest and highest concentration) of the optimized solutes (0.2 mL, 0.1 mL, 0.01 mL). The four different piper species were extracted in triplicate using the lowest-optimized-highest solute concentration to evaluate the effect of increasing or decreasing concentrations on PPN amount.

### 2.6 Statistical analysis

#### 2.6.1 Paired sample *t*-test

The statistical analysis used the SPSS (Statistical Package for Social Sciences) software V 27.0. The data for the two models (solutes vs PPN amount, and pepper species vs PPN amount) were entered into the SPSS file, and the paired *t*-test was employed to sort significant differences for the two models at *P* < 0.05.

## 3 Results

### 3.1 Green UPLCMSMS method development and validation

The chromatographic conditions developed for PPN separation were: mobile phase consisting of A (H_2_O) and B (EtOH) with an isocratic elution of 98% (B), flow rate of 0.5 mL/min, injection volume of 5 μL, column oven temperature of 40°C, and a runtime of 2 min for PPN with a retention time of 1.3 min. The column used for separation was Thermo Scientific Hypersil GOLD™ C18 (50 × 4.0 mm, 3 μm). The validation for the developed method resulted in an accuracy of 95–105 ± 14.24% with an r^2^-value of 0.99 in the linearity range of 0.01–500 ppb for PPN. The LOD and LOQ for the method were in the range of 0.07–10.3 ± 3.53 ppb and 0.2–31.21 ± 10.69 ppb, respectively. The validation data was generated and recorded from the in-built Shimadzu software for UPLCMSMS-8050. The data for the regression equation and validation parameters are shown in Table 1.

**Fig. 1:**
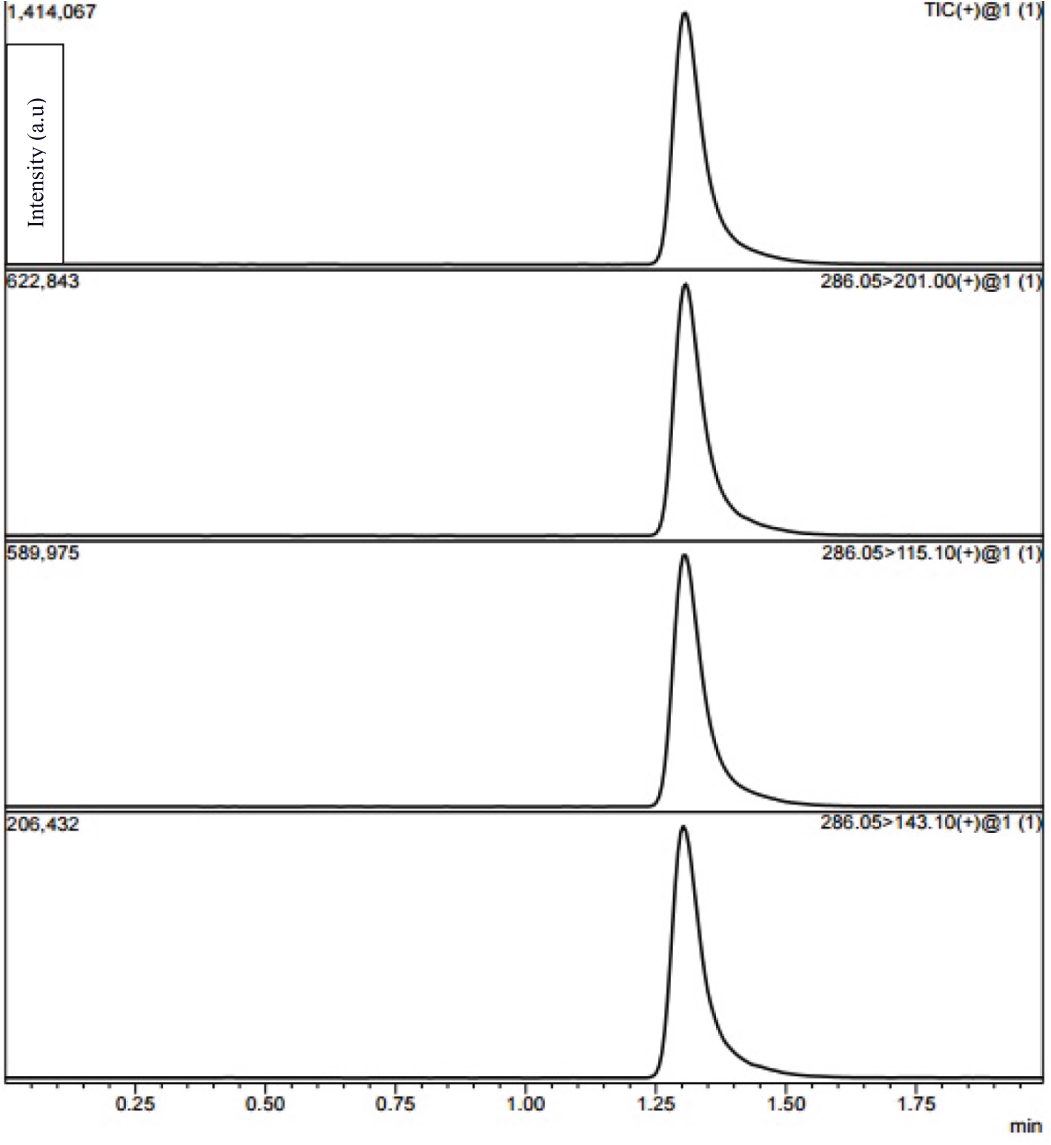
UPLCMSMS chromatogram for PPN showing the fragments at respective retention time.

**Fig. 2:**
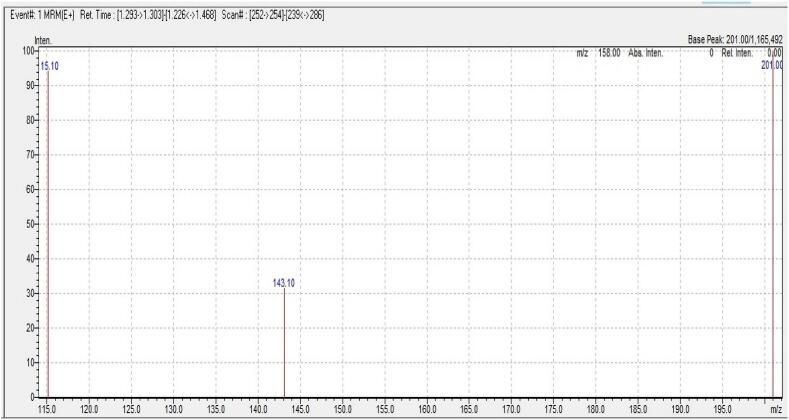
Fragmentation pattern for the ppn molecule at esi (+mode) presenting the base peak 201 *m*/*z*.

**Fig. 3:**
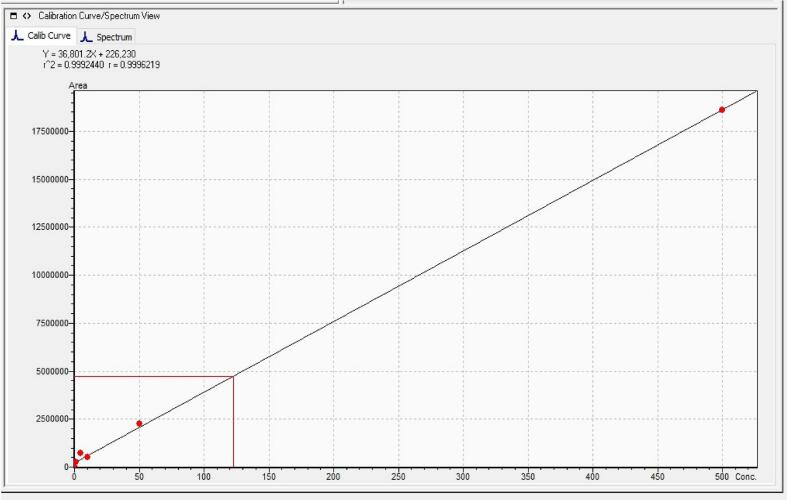
Calibration curve for ppn with regression equation and r^2^-value.

The MS conditions for the PPN fragmentation were: ESI (+ve mode), mobile phase with 70%EtOH, dwell time of 100msec, and event time of 0.309sec. The fragmentation pattern for PPN resulted in a pattern (*m*/*z*) of 286.05 → 201.0 → 143.10 → 115.10 with collision energies of −20, −30.9, and −50 for each fragment, respectively. The details regarding the gas flow for nebulization, heating, and drying, along with the temperatures for interface, DL, and heat block, are shown in Table 1. The chromatogram for PPN separation and fragmentation to the respective fragments is shown in Fig. 1, Fig. 2, the graph for the CC points with r^2^-value is shown in Fig. 3.

### 3.2 Solutes solubility and selection

#### 3.2.1 Finalization of the solute concentrations

To determine the appropriate concentrations of the solutes, a range of values was evaluated for each one, using a series of solubility and dilution tests. The concentrations that exhibited the highest solubility were chosen and further examined at two additional, higher concentration levels to encompass the complete solubility range. The final three concentration levels selected for each solute, labeled as codes 1–3, are presented in Table 2.

#### 3.2.2 Solutes optimal concentrations

The optimal concentration for the individual solutes observed was: 0.5 mL (T20), 0.1 mL (T80), 0.01 mL (L19), 0.01 mL (L21), 0.001 mL (LG), 100 mg (CD), 10 mg (PAA), 0.001 mL (EO), 1 g (PVA), 100 mg (SA), 0.1 mL (PF), 0.5 mL (TX), 50 mg (SDS), and 0.5 g (PEG). The data for the reference value, solute concentration range studied, and the optimal concentrations finalized are given in detail in Table 2.

### 3.3 UPLCMSMS analysis for PPN in fourteen optimized solutes

The solubility phenomenon for PPN exhibited a clear solution at a concentration of 1 mg/mL. The analytical method revealed the maximal PPN concentration extracted in the aqueous solutes of PF (4235.44 ppb) and EO (3405.24 ppb). The order for solutes with PPN concentration > 100 ppb was: PAA (3379.11) > SA (901.48) > SDS (168.52) > PEG (100.56). The amount of PPN in the aqueous-PPN extract was 645.34 ppb. The PPN quantification data for the fourteen different solutes are provided in Table 3.

**Table 3:** UPLCMSMS analysis for the solutes showing the solubility for PPN (ppb).

S#	**Solutes**	**Code**	**Optimum concentration**	**PPN/excipient ratio tested**	**PPN/excipient ratio finalized**	**PPN** **(ppb)**
1	Tween-20	T20	0.5 mL	1–5 mg/mL	1 mg/mL	24.59 ± 3.5
2	Tween-80	T80	0.1 mL			15.42 ± 4.7
3	Labrafil-M (1944-CS)	L19	0.01 mL			13.13 ± 4.1
4	Labrafil-M (2125-CS)	L21	0.01 mL			87.30 ± 6.3
5	Lauroglycol (FCC-1)	LG	0.001 mL			31.05 ± 7.7
6	Beta-cyclodextrin	CD	100 mg			21.17 ± 5.8
7	Polyacrylic acid	PAA	10 mg			3379.11 ± 29.8
8	Ethyl oleate	EO	0.001 mL			3405.24 ± 32.5
9	Polyvinyl alcohol (MW-25000)	PVA	1 g			54.69 ± 5.7
10	Sodium alginate	SA	100 mg			901.48 ± 22.1
11	Pluronic (F-68)	PF	0.1 mL			4235.33 ± 46.23
12	Triton (X-100)	TX	0.5 mL			28.10 ± 4.3
13	Sodium dodecyl sulfate	SDS	50 mg			168.52 ± 12.5
14	Poly ethylene glycol (MW-2000)	PEG	0.5 g			100.56 ± 11.4
	Water	W	−		1 mg/mL	645.34 ± 34.5

### 3.4 Two-level optimization for the PF and EO vs PPN concentration

#### 3.4.1 Level-I concentrations for PPN solubility

The solutes of PF and EO studied at three concentrations (Conc-I, Conc-II, Conc-III) of Level-I resulted in the PPN amount to be: PF Concentration-I (40) < -II (4135.88) < -III (5290.79), whereas, for EO, the ascending order of PPN extraction was: EO Concentration-I (821.07) < -II (2079.01) < -III (1912.11). The data for Level-I concentrations studied for PPN solubility are shown in Table 4.

**Table 4:** Two-level dilution with extraction of the ppn from the pepper species using pf aqueous solutes.

**Solutes**	**Level-I**	**Concentration optimization**	**PPN-amount (ppb)**	**Ultrasonic extraction parameters**
**PF**	Conc-I	0.2 mL	40 ± 6.8	Amplitude (40%); pulse (40 s:5s); time (3 min); sample amount (200 mg); optimized aqueous PF-solutes volume (10 mL)
	Conc-II	0.1 mL	4135.88 ± 65.8	
	Conc-III	0.05 mL	5290.79 ± 78.2	
**EO**	Conc-I	0.005 mL	821.07 ± 38.2	
	Conc-II	0.001 mL	2079.01 ± 45.6	
	Conc-III	0.0005 mL	1912.11 ± 24.1	
**Level-II optimization for PF**				
**PF**	Conc-IV	0.005 mL	43.86 ± 8.3	
	Conc-V	0.001 mL	34.65 ± 5.6	
	Conc-VI	0.0005 mL	20.02 ± 7.5	
**U**A**E for Pepper species**				
PN1	Level-1	0.2 mL	27.39 ± 9.1	
PN2	Level-2	0.1 mL	50.34 ± 12.3	
PN3	Level-3	0.01 mL	83.43 ± 24.3	
PL1	Level-1	0.2 mL	14.43 ± 9.1	
PL2	Level-2	0.1 mL	21.50 ± 12.8	
PL3	Level-3	0.01 mL	22.98 ± 9.4	
PC1	Level-1	0.2 mL	1.14 ± 6.4	
PC2	Level-2	0.1 mL	1.77 ± 8.3	
PC3	Level-3	0.01 mL	2.80 ± 6.2	
PW1	Level-1	0.2 mL	39.30 ± 15.4	
PW2	Level-2	0.1 mL	118.65 ± 29.8	
PW3	Level-3	0.01 mL	147.20 ± 33.2	

#### 3.4.2 Level-I concentrations for PPN solubility

The PF solutes studied at three further diluted concentration levels (Conc-IV, Conc-V, Conc-VI) exhibited the PPN concentration (ppb) extracted: PF-IV (43.86) > PF-V (34.65) > PF-VI (20.02). The data regarding the two-level solute optimization at lower and higher concentrations of the optimal value is provided in Table 4.

### 3.5 USE for PPN using the optimal solute concentration

The USE for PPN at the three levels of the PF-solutes concentrations revealed a higher amount of PPN (ppb) for PW, followed by PN > PL > PC. The data for the three levels of PF-solutes exhibited more amount of PPN extraction for the lowest concentration of PF (ppb) used: PW [PW3(147.20) > PW2 (118.65) > PW1 (39.30)], PN [PN3 (83.43) > PN2 (50.34) > PN1 (27.39)], PL [PL3 (22.98) > PL2 (21.50) > PL1 (14.43)], and PC [PC3 (2.80) > PC2 (1.77) > PC1 (1.14)].

**Fig. 4:**
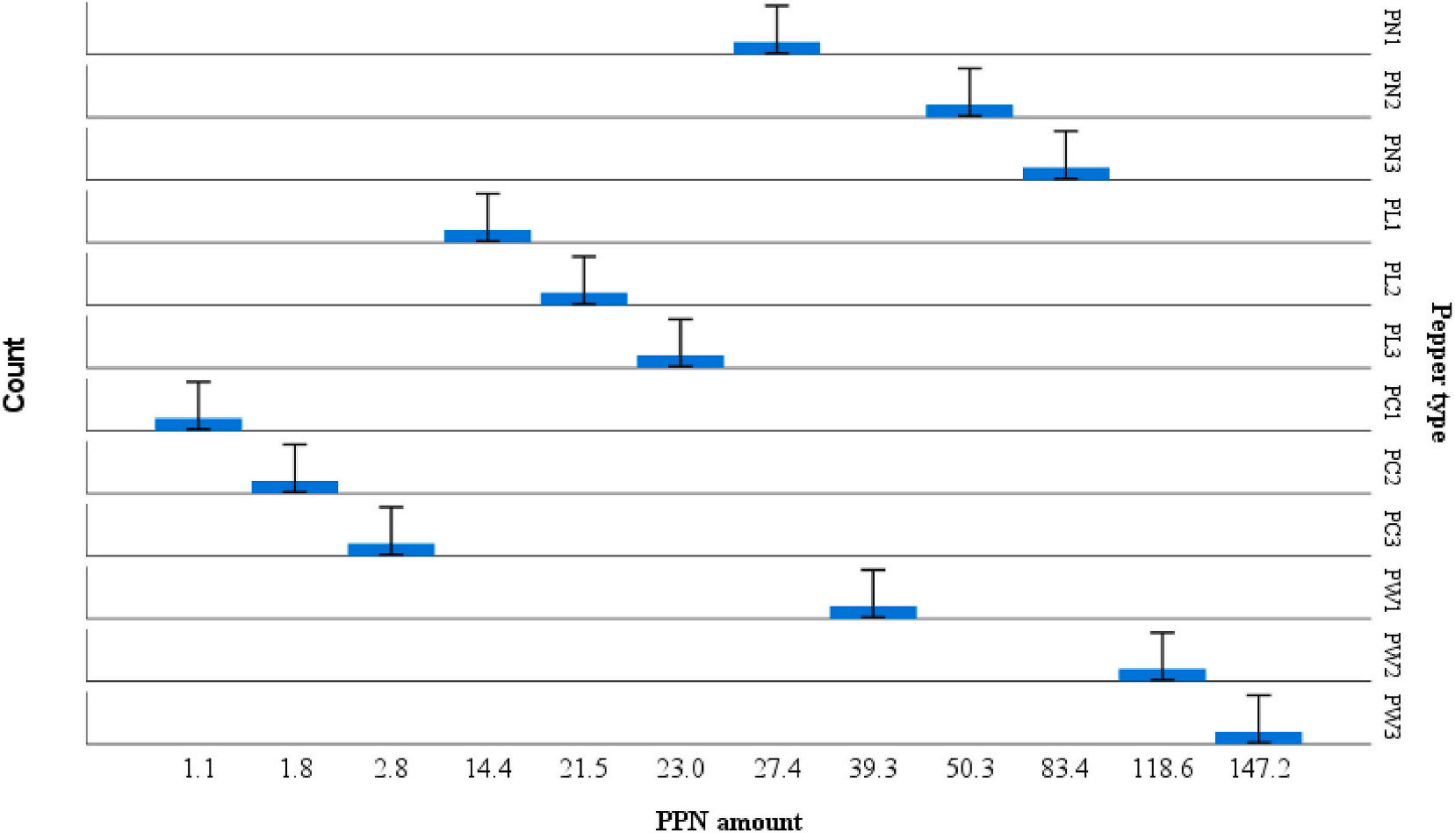
The USE-yield for ppn amount from different pepper species using solutes.

The order for PPN extraction vs the three levels of PF-solutes constructed the descending order of: PW3 (147.20) > PN3 (83.43) > PL3 (22.98) > PC3 (2.80). The details regarding USE extraction for PPN in the four different species of piper at three different PF-levels are shown in Table 4 and Fig. 4.

### 3.6 Paired sample *t*-test

#### 3.6.1 Model-I (Paired sample test for PPN vs solutes)

The paired sample *t*-test for model I revealed: *M* = 874.04, SD = 1482.60, compared to the calculated mean *M* = 866.07, SD = 141.82, exhibiting a significant difference at *t(14)* = 2.264 with 95%CI (45.47 – 1686.68), *P <* 0.05. The test was seen with a large sample effect size of 0.58 (Cohen’s d) and 0.57 (Hedges’ correction).

#### 3.6.2 Model-II (Paired sample test for PPN vs pepper species)

The analysis using paired sample *t*-test for Model II revealed: *M* = 44.24, SD = 47.92 compared to the calculated mean of *M* = 37.75, SD = 46.63, revealing a significant difference at *t (11)* = 2.804 with 95%CI (8.12 – 67.37), *P <* 0.05. The effect size for the sample observed was large at 0.81 (Cohen’s d) and 0.78 (Hedges’ correction). The details regarding the paired sample *t*-test for both models are given in Table 5.

**Table 5:** Paired sample*t*-test for the solutes vs PPN solubility behavior and pepper species vs PPN amount: mean ± SD with 95% confidence interval (CI) and *P*-value (0.05), effect size for Cohen’s d and Hedges’ correction.

**Model I: Paired test for solutes vs PPN amount**						
		**Mean**	**N**	**SD**	**Std. Error Mean**	
**Pair 1**	**PPN amount**	874.07	15	1482.60	382.81	
	**Solutes**	8.00	15	4.47	1.15	
**PPN amount-Solutes**	**Mean**	**Std. Dev**	**95% CI**		**t**	***P***
			**Lower**	**Upper**		
	866.07	1418.2	45.47	1686.68	2.264	0.04
***Paired samples Effect sizes***						
**Pair 1**			**Standardizer**	**Point estimate**	**95% CI**	
					**Lower**	**Upper**
**PPN amount-Solutes**		**Cohen's d**	1481.82	0.58	0.03	1.13
		**Hedges' correction**	1523.04	0.57	0.03	1.10
**Model II: Paired test for pepper species vs PPN amount**						
		**Mean**	**N**	**SD**	**Std. Error Mean**	
**Pair 1**	**PPN amount**	44.24	12	47.92	13.83	
	**Pepper species**	6.50	12	3.61	1.04	
**PPN amount- pepper species**	**Mean**	**Std. Dev**	**95% CI**		**t**	***P***
			**Lower**	**Upper**		
	37.75	46.63	8.12	67.37	2.80	0.01
***Paired Samples Effect Sizes***						
**Pair 1**			**Standardizer**	**Point Estimate**	**95% CI**	
					**Lower**	**Upper**
**PPN amount- pepper species**		**Cohen's d**	46.63	0.81	0.14	1.45
		**Hedges' correction**	48.29	0.78	0.13	1.40

## 4 Discussion

The study undertakes the challenge of enhancing the aqueous solubility of PPN through a sustainable approach in which solutes in the form of various polymers, surfactants, and solubilizing agents, along with green solvents, are utilized. The proposed method aims to provide a sustainable method in order to abolish the need for the traditional toxic solvents in extraction. This addresses the eco- and health-related concerns associated with the use of the mentioned toxic solvents. To execute the research idea, a thorough literature review was conducted to gather information on the most widely used solutes and their respective CMC values employed for natural products extraction [[25], [26], [27], [28], [29], [30], [31], [32], [33]]. Fourteen solubilizing agents, T20, T80, L19, L21, LG, CD, PAA, EO, PVA, SA, PF, TX, SDS, and PEG were chosen by screening the literature, and their capacity in increasing PPN solubility in water was investigated. When using CMC values taken from the literature, the solutions were not clear at all, so the lowest values were used and diluted serially. The prepared solutions were vortexed for 5 min, sonicated for 15–20 min, and centrifuged for 10 min at 10,000 rpm to obtain a clear solution. Then, five serial dilutions of each solute were prepared, and PPN was dissolved (1–3 mg/ml). The initial choice of solute and its concentration was determined visually (compared to the solution of PPN in EtOH), and the lowest concentration that gave a clear solution was chosen, optimized for a final concentration, and the highest solubility was calculated. All the solute systems at the end gave a PPN solution with the highest solubility up to 1 mg/ml at the lowest concentration possible of a solute. The quantitative measurements for PPN in these samples were done using UPLCMSMS.

An in-house green, economical, eco-, and human-friendly analytical method was developed and validated. The UPLCMSMS resulted in a shorter runtime analysis for PPN with an RT of 1.3 min using the green solvents of EtOH and H_2_O, with MS fragmentation resulting in a base peak of 201 *m*/*z*. The developed method was validated as per the ICH guidelines, showing a high accuracy of 95–105% with LOD and LOQ of 0.07–10.3 and 0.2–31.21 ppb, respectively. Herein, a fast, green, and sensitive UPLCMSMS method for PPN determination was established. The fourteen solubilizers were pre-optimized and prepared in the lowest effective concentrations. The solubilization of PPN was assessed, and then 1 mg/ml was chosen as the final concentration since it was much better. All the solubilizers were prepared in water at the lowest effective concentration and mixed with the PPN standard solution (1 mg/ml), vortexed, sonicated, and centrifuged to remove insoluble material. The upper layer from the centrifuged solutions was withdrawn, diluted with distilled water, syringe filtered, and subjected to UPLCMSMS analysis for PPN quantification. In addition to the dissolution of PPN in the fourteen solutes, an aqueous PPN solution was also prepared to serve as a standard for measuring the comparative solubility behavior of PPN in aqueous medium. The UPLCMSMS results exhibited an amount of 645.34 ppb for aqueous PPN, whereas the aqueous solutes with comparatively more solubility for PPN were noted to be: PF (4235.33 ppb) > EO (3405.24 ppb) > PAA (3379.11 ppb) > SA (901.48 ppb). This indicates a sevenfold (7x) and fivefold (5x) increase in PPN solubility in PF and EO, respectively, compared to water. The remaining aqueous solutes were seen with a lower solubility profile for PPN. Both PF and EO were chosen for the investigation of PPN solubility based on optimal performance. The range of concentrations was determined using both solutes at three graded concentrations (Conc I-III), high, optimum, and low concentrations, using a PPN amount of 1 mg/mL. PF exhibited increasing solution solubility as the concentration of dilution decreased, while EO showed very poor performance and was discarded. To optimize the selection, PF was analyzed at three further diluted concentrations, ranging from higher, optimum, and lower concentrations (Conc IV-VI). The solubilization decreased at concentrations beyond the optimum, suggesting Conc-III as the optimum concentration for increasing the PPN solubility. The PF presence may induce a crystalline-to-amorphous transition of the drug molecules within its micelles, further increasing the dissolution rates [26]. The authors suggest a similar mechanism for the ∼ 7-fold PPN-solubility enhancement observed with the use of PF. In addition, the biocompatibility and stabilizing properties favor PF as a valuable excipient for green pharmaceutical formulations. [34] In detail, the triblock copolymer of PF enhances PPN solubility via formation of micelles where the hydrophobic PPO, i.e., Poly(propylene oxide) core, entraps PPN, whereas the hydrophilic PEO, i.e., Poly(ethylene oxide) chains, provide aqueous stabilization. This micellar encapsulation promotes a crystalline-to-amorphous transition, protects PPN from precipitation, and achieves efficient solubilization at low concentrations due to PF’s low critical micelle concentration [35]. It is suggested that improved solubility of PPN in the presence of amphiphilic solutes like PF and EO is because these solutes may establish hydrophobic environments in aqueous media that will incorporate lipophilic substances. PF is known to exhibit micellar behavior, with such hydrophobic molecules forming stable encapsulated complexes. The structure of PF, containing a hydrophobic core composed of PPO, allows encapsulating hydrophobic molecules and improves their solubility in water. [[32], [36], [37]] The increase in solubility seen at sub-CMC concentrations points to a role of pre-micellar aggregation in this phenomenon. The hydrophobic domains of pre-micellar structures might be more exposed. This is favored for solute–solute interactions and can facilitate aqueous stabilization of PPN.

In order to study the application of the optimized system, the extraction of PPN from different species of peppers, i.e., *Piper nigrum* (white and black), *Piper longum*, and *Piper cubeba*, was evaluated. An ultrasound-assisted green procedure was used for extraction to determine both efficacy and fitness for relative comparison of quality between species. [24] The USE provides shorter extraction time, less solvent, and a smaller sample size, and good repeatability in natural products analysis. The PPN content in these pepper species was in the range of 27.39–83.43 ppb (black) and 39.30–147.20 ppb (white) for the variety of *Piper nigrum*, 14.43–22.98 ppb (*Piper longum*), 1.14–2.80 ppb (*Piper cubeba*), showing efficient extraction in an aqueous system. The descending order of PPN was: white pepper > black pepper > long pepper > cubeb pepper, consistent with the earlier studies of more PPN in *P. nigrum*. [[38], [39]]. The higher purity sometimes observed in white pepper extracts may stem from the removal of the pericarp and concentration of internal PPN, aligning with our results [[38], [40]]. The result obtained agrees well with the literature report, and it also reveals that the developed system with an aqueous-solute system is feasible for extracting PPN. Interestingly, the extraction efficiency decreased as solute concentration increased, and the highest yield was found at mid-dilution concentration (Conc-II) in all the tested species of pepper. This clearly shows that maximum solubilization doesn't happen at high solute concentration; instead, it depends upon an optimum sub-CMC range. This is reported to be due to the formation of pre-micellar aggregation, wherein sub-CMC structures offer a hydrophobic environment suitable for the solubilization of lipophilic solutes. Similar findings were also observed in the case of solubilization of hydrocarbons, flavonoids, etc. [[41], [42]] The higher efficiency under sonication is due to the acoustic cavitation effect, in which local high-pressure and high-temperature microzones lead to the fracture of cell walls, allowing and enabling transfer of intracellular matter into the extraction solution. [[15], [43]] In contrast to previously documented USE-based methodologies for PPN extraction, which frequently necessitate the use of organic solvents and extended extraction periods, the present approach achieves efficient extraction within a significantly shorter duration while concurrently utilizing an aqueous solute system. [9], [10], [11], [12], [13], [14] This increase in extraction efficiency can be attributed to a cooperative mechanism of ultrasonic-induced matrix disintegration and solute-induced solubilization. Unlike hydrotropic systems, which rely on a large excess of the solute for their efficacy, the present system uses the solute at relatively low concentrations, thereby enabling its effective molecular-level interaction to achieve solubilization of PPN, as shown by quantitative analysis via UPLCMSMS. [8] Although USE methods [9], [10], [11], [12], [13], [14] are frequently employed to improve mass transfer efficiency, the majority of these studies employ organic solvents and extended periods. However, in this work, efficient recovery of PPN was observed utilizing the aqueous system with the addition of low concentrations of solute and taking advantage of the amphiphilic effect and sub-CMC aggregation. The statistical model of paired *t*-test for solutes vs PPN amount, and pepper species vs PPN amount presented significant differences for the PPN amount extracted by various solutes in different types of pepper species. The mean differences were significant at *P < 0.05*. The results for the sample effect sizes for the solutes vs PPN amount, and pepper species vs PPN amount were moderate to high, indicating a pronounced effect on PPN amount with the change in solute nature as well as the type of pepper species.

The developed aqueous-solute model is an effective tool for PPN extraction from pepper species in substitution for toxic organic solvents. The method is eco-friendly for having both reduced toxicity and improved sustainability, operating in an aqueous medium under sonication, contributing to improved efficiency and shorter extraction time. The results produced by the Analytical Eco-Scale method showed good greenness (score = 83), mainly due to the application of water and safe amphiphiles as solvents and a short sonication extraction time that lowers environmental costs and energy consumption. The minor penalties resulted from the restricted usage of organic solvents in chromatographic analysis and in the energy necessary for UPLCMSMS detection. The results demonstrate greener extraction as per the greenness scales. [[44], [45]] The calculation for the greenness score of the method is shown in Table 6. The model needs to be validated for the extraction of PPN from other metrics, including food products, herbal and dietary supplements, and pharmaceutical products. This will pave the way for the applications of a short, green, sensitive, efficient, and reproducible method in PPN extraction and analysis.

**Table 6:** The greenness profile calculated based on anastas and gałuszka, and płotka-wasylka green analytical chemistry metrics.

**Parameter**	**Assessment**	**Penalty Points**
Solvent	Water	0
Solute	PF, EO (Low toxicity)	4
Solvent for UPLCMSMS	Less volume of acetonitrile	6
Energy consumption	UAE (3 min process with low energy and short duration)	2
Instrumentation	UPLCMSMS with moderate energy usage	2
Waste generated	Low volume with mostly aqueous phase	3
Occupational hazard	Low (no toxic solvents in extraction)	0
*Total penalty points*		17
*Greenness scale points*		83

## 5 Conclusion

This research work describes a green and efficient aqueous solute-based system for extracting PPN from pepper species. Amongst the solubilizers examined, PF gave the best performance with nearly 7-fold solubility enhancement compared to aqueous systems. The quantification using UPLCMSMS allows for highly selective and accurate analysis of the extraction efficiencies of the different pepper species. The aqueous solute system may serve as an efficient substitute for the conventional organic solvent-based extraction system. The aqueous solute-based extraction system is a safer and cheaper alternative to the conventional methods. The system provides the necessary solubilization enhancement; the detailed mechanism of solute-induced solubility enhancement in aqueous medium needs further study. The main novelty of the current work is to create an aqueous system with an engineered solvent by adding solutes, which follows green chemistry principles, and it can be used as a scalable platform for the extraction of poorly water-soluble phytochemicals and possibly other lipophilic natural products.

## Funding source

This research work was funded by Umm Al-Qura University, Saudi Arabia under grant number: 26UQU4280107GSSR02.

## Consent to publish

The authors in this study agree to publish the data in its current format.

## CRediT authorship contribution statement

Rizwan Ahmad (study design); Deema Alaswad, Ayat Jaffer Alshehab, Ahad Alsulais, Wala Alsulais (collection of samples, experimental work, data collection, analysis), Rizwan Ahmad, Mohammed Aldholmi (UPLCMSMS method development and validation, samples quantification, calibration curve development); Rizwan Ahmad, Aljawharah Alqathama (data execution and SPSS analysis), Fatimah Taiseer Alghorab, Hawraa Ayesha Alibrahim (Literature review and introduction write-up); Deema Alaswad, Ayat Jaffer Alshehab, Ahad Alsulais, Wala Alsulais (discussion write-up, final review); Rizwan Ahmad, Mohammed Aldholmi, Aljawharah Alqathama (critical review of the manuscript with final approval).

## Declaration of Competing Interest

The authors declare that they have no known competing financial interests or personal relationships that could have appeared to influence the work reported in this paper.
